# From Dysbiosis to Disease: The Microbiome’s Influence on Uveitis Pathogenesis

**DOI:** 10.3390/microorganisms13020271

**Published:** 2025-01-25

**Authors:** Priya D. Samalia, Jahnvee Solanki, Joseph Kam, Lize Angelo, Rachael L. Niederer

**Affiliations:** 1 Health New Zealand Auckland, Auckland 1051, New Zealand; 2 Department of Medicine, University of Otago, Dunedin 9016, New Zealand; 3 Department of Ophthalmology, University of Auckland, Auckland 1010, New Zealand

**Keywords:** microbiome, uveitis, dysbiosis, T regulatory cells, T effector cells

## Abstract

The microbiome, comprising the diverse microbial communities inhabiting the human body, has emerged as a critical factor in regulating immune function and inflammation. The relationship between the microbiome and uveitis represents a promising frontier in ophthalmological research, with the microbiome increasingly implicated in disease onset and progression. Research has predominantly focused on the gut microbiome, with animal studies providing evidence that dysbiosis is a key factor in autoimmunity. As the understanding of the microbiome increases, so does the potential for developing innovative treatments that leverage the microbiome’s impact on immune and inflammatory processes. Future research will be crucial for deciphering the complexities of the interaction between the microbiome and immune system and for creating effective microbiome-based therapies for those with uveitis. Incorporating microbiome research into clinical practice could transform how uveitis is managed, leading to better and more individualized approaches for management. This review discusses the current understanding of the microbiome–uveitis axis, the promise of microbiome-based diagnostics and therapeutics, and the critical need for large-scale, longitudinal studies. Unlocking the potential of microbiome-targeted approaches may revolutionize the management of uveitis and other inflammatory diseases.

## 1. Introduction

**Figure 1:**
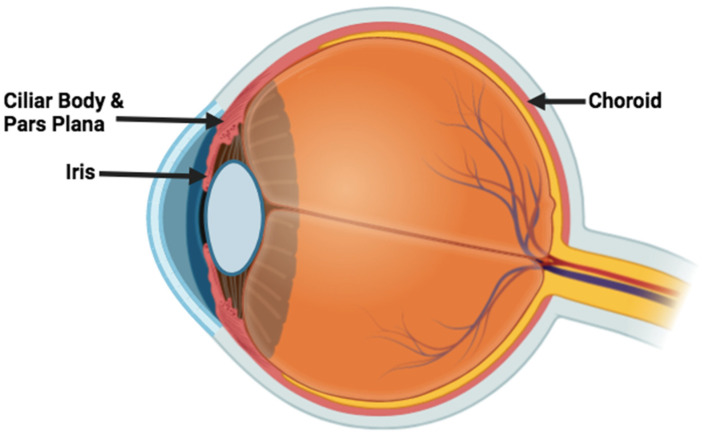
The uveal tract. Figure generated using biorender.com.

Uveitis is a group of inflammatory conditions affecting the uveal tract (Figure 1), which consists of the iris, ciliary body, and choroid. It can also involve adjacent structures such as the retina, vitreous, and optic nerve. Uveitis is a leading cause of vision impairment and blindness globally [1,2]. It manifests with a range of symptoms, including eye redness, pain, light sensitivity, floaters, and blurred vision. This heterogenous group of inflammatory eye diseases may arise from infections, autoimmune responses, systemic conditions, ophthalmologic disorders, drug-induced reactions, or masquerade syndromes [3]. The condition typically affects young, working-aged individuals [4].

**Figure 2:**
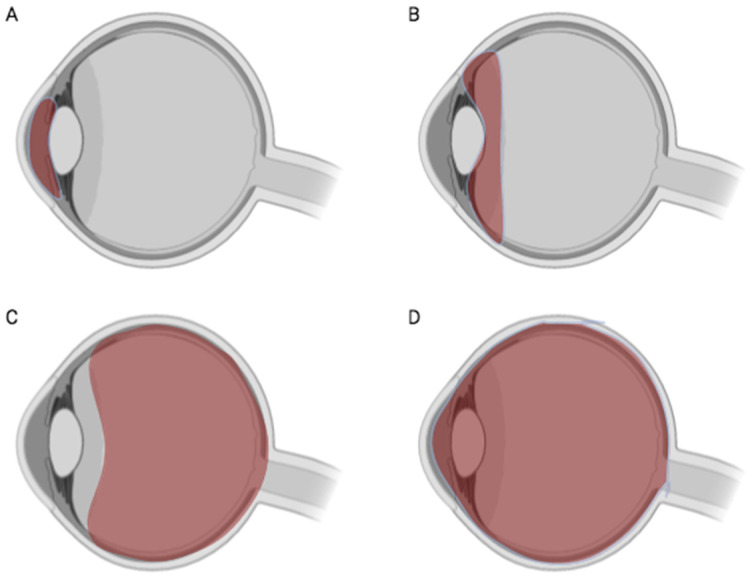
Anatomic classifications of uveitis. (**A**) Anterior uveitis primarily affects the iris, (**B**) intermediate uveitis targets the ciliary body, (**C**) posterior uveitis involves the retina and choroid, and (**D**) panuveitis involves the entire uveal tract. Figure generated using biorender.com.

Uveitis can be classified anatomically into four categories based on the anatomical site of inflammation: anterior, intermediate, posterior, and panuveitis [5] (Table 1, Figure 2). Anterior uveitis, the most common form, primarily affects the iris. Intermediate uveitis involves inflammation of the intermediate portion of the uveal tract, including the ciliary body. Posterior uveitis affects the retina and choroid, while panuveitis refers to inflammation that involves the entire uveal tract.

Chronic and recurrent inflammation, accompanied by immune cell infiltration, can lead to uveal damage and sight-threating complications. Common complications of uveitis include cystoid macular edema, glaucoma, cataract, and posterior synechiae [6,7]. Uveitis accounts for 10–15% of blindness in the developed world [1]. Given its prevalence in young, working-age individuals, uveitis imposes a substantial burden on both affected individuals and society. The economic cost of vision loss has been estimated at USD 134.2 billion, with reduced labor force participation accounting for USD 16.2 billion [2]. Beyond the financial costs, vision impairment significantly reduces quality of life, leads to poor psychosocial outcomes, and increases the risk of injuries and premature mortality [8].

The treatment of uveitis focuses on suppressing inflammation and preventing further loss of sight. While corticosteroids remain the cornerstone of therapy, there is increasing interest in alternative therapeutic strategies. Among these, microbiome modulation has emerged as a potential adjunct to conventional treatments [9,10].

The onset and progression of uveitis are influenced by a complex interplay of genetic, immunological, and infectious factors [3]. Generally, uveitis develops when there is a breakdown in immune tolerance against endogenous antigens. This triggers the production of autoantibodies, dysregulation of effector cells (such as Th1 and Th17), and a disruption of regulatory T cells (Tregs). This immune imbalance leads to ocular infiltration of T lymphocytes and macrophages [9,10]. Cytokine pathways play a crucial role in driving inflammation, with elevated levels of pro-inflammatory cytokines such as IL-6, IL-17, and IL-23, and tumor necrosis factor-alpha found in both the blood and ocular fluid of patients with uveitis [11].

The microbiome may play a crucial role in the development of autoimmune responses by acting as a source of antigens and antigen-specific T cells [10]. Recent studies have highlighted the microbiome’s involvement in autoimmune disease, including uveitis, suggesting its potential as a target for novel therapies. This narrative review synthesizes the current state of knowledge on the relationship between the microbiome and uveitis, highlighting emerging themes, therapeutic avenues, and identifying areas requiring further research to better understand how microbiome-based therapies can be integrated into clinical practice for managing uveitis.

A thorough literature search was performed across multiple databases, including MEDLINE via Ovid, PubMed, Embase, and the Cochrane Database of Systemic Reviews, using a combination of keywords such as “microbiome and uveitis”, “gut microbiome in autoimmune disease”, “oral microbiome and ocular inflammation”, “systemic disease associated with uveitis”, and “probiotics and uveitis treatment”. Studies were included if they focused on microbiome alterations in uveitis, its systemic associations (including ankylosing spondylitis, inflammatory bowel disease, psoriasis, sarcoidosis, Vogt–Koyanagi–Harada disease, and Behçet’s disease), and therapies targeting the microbiome. Articles were selected based on their relevance to understanding microbiome mechanisms in uveitis and the therapeutic implications of microbiome modulation. The reference list of articles and related articles was used to screen additional studies.

The literature was classified into key thematic areas: (1) mechanisms of microbiome influence on uveitis, which includes antigen mimicry, loss of intestinal-immune homeostasis, reduction in anti-inflammatory microbial metabolites, and loss of intestinal barrier integrity; (2) systemic diseases associated with uveitis (such as ankylosing spondylitis, inflammatory bowel disease, and Behçet’s disease), with discussion of how dysbiosis contributes to the development of disease; (3) evidence of dysbiosis in uveitis including experimental animal models and human studies; (4) technological advances in microbiome research, focusing on the application of next-generation sequencing (NGS) and metagenomics to better understand microbial communities and their interactions with immune responses in uveitis; and (5) the therapeutic potential of microbiome modulation, which examines the role of probiotics, prebiotics, antibiotics, fecal microbiota transplant (FMT), and immunosuppressive therapies in managing uveitis through microbiome manipulation.

## 2. The Human Microbiome

The human microbiome consists of trillions of microorganisms, including bacteria, viruses, fungi, and protozoa, inhabiting various body sites such as the gut, skin, mouth, and eyes. When the microbiome maintains a symbiotic relationship with the host, homeostasis is preserved. This is essential for the proper functioning of the immune system and the maintenance of health. Microbiome composition varies significantly across different anatomical sites (skin, nostrils, mouth, intestine, eye, etc.), reflecting the unique environmental conditions and functions of each location [10]. Commonly found microbial taxa in different body sites are liste in Supplementary Table S1.

The composition of the microbiome can be influenced by host factors such as age and gender, as well as environmental factors, such as diet and medication. Of these, diet is considered to have the most significant impact in determining microbiota composition [12]. However, other influences, such as chronic stress, circadian rhythms, medication use, toxin exposure, colonization by external microbes, and various diseases, also play a role in shaping microbiome composition [13,14]. 

Emerging research suggests that an imbalance in the human microbiota, known as dysbiosis, may contribute to the onset and severity of autoimmune diseases, including uveitis [13,15]. The gut–eye axis, where gut-derived immune cells or microbial products influence eye inflammation, appears to be a key mechanism in this process. In uveitis, specific organisms associated with dysbiosis include *Malassezia*, *Candida*, and *Aspergillus gracillis*, while *Facealibacterium*, *Lachnospira*, *Ruminococcus*, *Blautia*, and *Roseburia* are reduced [16].

## 3. Gut Microbiome

The human gut microbiome is the largest and the most diverse microbial community in the body, comprising approximately 10^14^ microorganisms [17]. More than 90% of the microbiome is located in the gut [18]. The dominant microbial phyla in both humans and mice are *Firmicutes* and *Bacteroidetes*, followed by *Actinobacteria* and *Proteobacteria*, while *Fusobacteria* and *Verrucomicrobia* are less prevalent [19,20,21,22,23,24]. This microbial community is established at birth, primarily through maternal transmission, and evolves throughout life, maintaining a dynamic balance with the host’s immune system [25]. Infants delivered vaginally acquire microbiota dominated by maternal vaginal and gut bacteria, such as *Lactoacillus* and *Bifidobacterium* [26]. In contrast, those born by caesarean section are predominantly colonized by skin-associated microbes like *Staphylococcus*, which can lead to differences in microbiome development and potentially increase the risk of immune-related conditions, including allergies, asthma, and autoimmune diseases [13,26]. As individuals age, the microbiome continues to adapt, influenced by life changes and environmental factors. Early life exposures, such as breast feeding, influence microbiota composition by providing prebiotics and beneficial bacteria like *Bifidobacterium*, which support immune system development [27]. Later influences include diet, hormonal changes, and aging. High fiber diets support a diverse microbiota, while processed foods, antibiotics, and chronic stress can disrupt it, leading to dysbiosis and immune dysfunction [28,29].

The significance of the gut microbiome in immune function and inflammation was first highlighted in studies involving germ-free newborn mice, which showed significantly reduced levels of intra-epithelial lymphocytes and serum IgA, both essential components of the immune defence system [30]. These deficiencies were restored upon microbial colonization [30,31]. A subsequent study showed that the differentiation of Th17 cells in the lamina propria depends on the presence of segmented filamentous bacteria in the gut [32]. Beyond these local effects, immune responses to the gut microbiota are believed to play a central role in immune system development and may influence long-term susceptibility to infectious and autoimmune diseases [30].

## 4. Oral Microbiome

The oral microbiome consists of the microorganisms inhabiting the human oral cavity [33] and is the second largest microbiota, comprising over 700 species of bacteria [34]. The principle bacterial genera found in a healthy oral cavity are shown in Table 2. Commensal populations within the oral microbiome help regulate pathogenic species by preventing their adhesion to the mucosal surfaces. Pathogenicity occurs only when these protective commensals are bypassed, allowing pathogenic bacteria to invade, establish infections, and cause disease [35].

## 5. Eye Microbiome

The ocular microbiota refers to the diverse array of microorganisms present on or in the eye [9]. Common bacteria isolated from these sites of the eye are Gram-positive genera, including *coagulase-negative Staphylococcus*, *Streptococcus*, *Propionibacterium*, *Diphtheroid bacteria*, and *Micrococcus* [36]. Microbial density tends to be lowest in the tears and higher in the conjunctiva and eyelids [37].

## 6. Pathogenesis of Uveitis: The Microbiome Connection

Mechanisms of Autoimmune Uveitis Induced by the Gut Microbiome.

When combined with environmental and genetic factors, dysbiosis plays a significant role in the development of autoimmune diseases, including uveitis [31]. Dysbiosis may contribute to the development of uveitis through four primary mechanisms: antigen mimicry, loss of intestinal-immune system homeostasis, reduction in anti-inflammatory microbial metabolites, and loss of intestinal barrier integrity with increased intestinal permeability.

### 6.1. Antigen Mimicry

Antigen mimicry refers to a phenomenon where microbial antigens share structural similarities with the body’s own self-antigens. When the immune system mounts a response against the pathogen, it may inadvertently target the body’s own tissue if it fails to distinguish between the foreign antigen and the self-antigens. This cross-reactivity can lead to autoimmune disease. Antigen mimicry has been implicated in diseases linked to the human leukocyte antigen-B27 (HLA-B27) [38], where microbial species like *Chlamydia trachomatis*, *Campylobacter jejuni*, and genera like *Klebsiella*, *Salmonella*, *Yersinia*, and *Shigella* are involved [39,40].

**Figure 3:**
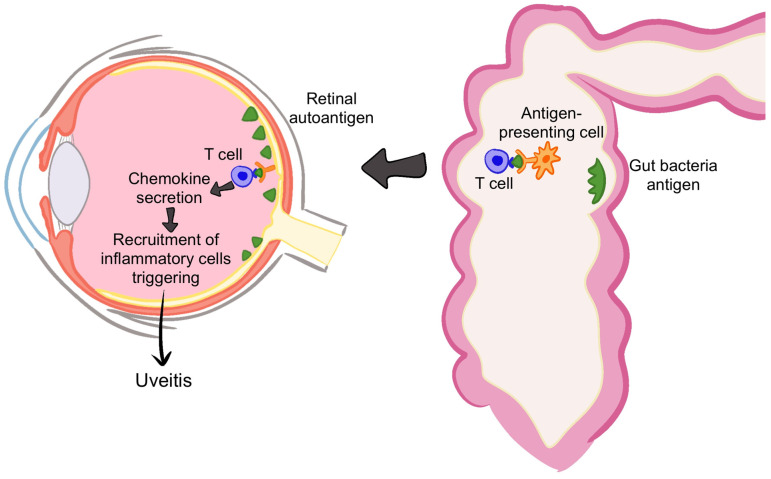
Antigen mimicry in the microbiome can lead to autoreactive T cells that cross ocular barriers, triggering intraocular inflammation. Image created with Procreate, Savage Interactive Pty Ltd., Version History: 5.3.5. Tasmania, Australia.

In the context of uveitis, gut microbiome antigens may play a role in the development of autoreactive T cells through antigen mimicry [41,42] (Figure 3). These autoreactive T cells can cross the blood–retina or blood–aqueous barrier, entering the eye and triggering intraocular inflammation [42]. Studies using animal models, such as mice with experimental autoimmune uveitis (EAU), have shown that disruption of the microbiota through antibiotics reduces uveitis severity and intestinal Th17 activation [43]. Furthermore, transferring T cells from microbiota-exposed mice to wild-type mice can induce uveitis, suggesting that microbial interactions contribute significantly to disease pathogenesis [43].

Additionally, antibodies targeting the retinal S-antigen have been implicated in autoimmune uveitis [41,42]. Peptides from *Escherichia coli*, which shares amino acid homology with a retinal S-antigen peptide, can induce cross-reactivity of T lymphocytes in vitro [44]. Similarly, mimotopes of retinal S-antigens derived from *Hepatitis B* virus, baboon virus, and *Rotavirus* have been shown to induce uveitis in Lewis rats after immunization [45].

While these studies lend support to the antigen mimicry hypothesis, the specific microbial antigens that trigger uveitis in humans have yet to be identified [42]. Identifying these triggers remains a critical challenge in understanding the precise mechanism by which microbial antigens contribute to autoimmune uveitis.

### 6.2. Loss of Intestinal-Immune System Homeostasis

**Figure 4:**
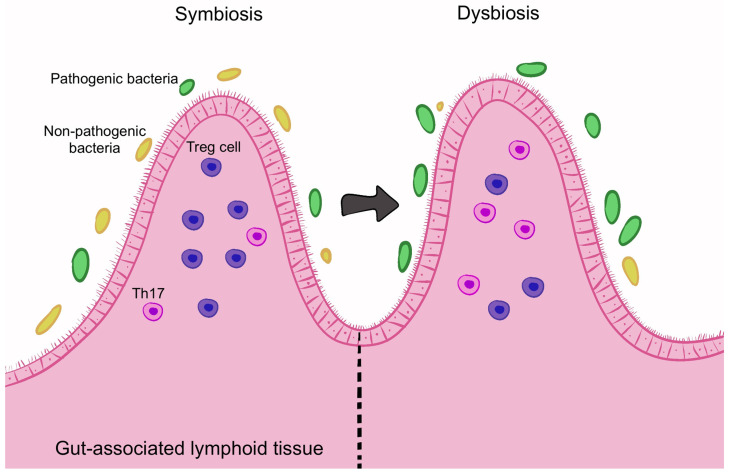
The gut microbiota regulates the balance between pro-inflammatory Th17 cells and Treg cells. Dysbiosis can increase Th17 cells and decrease Treg cells, triggering inflammation. Image created with Procreate, Savage Interactive Pty Ltd., Version History: 5.3.5.

The commensal microbiota plays a crucial role in regulating the balance between pro-inflammatory Th17 cells and inhibitory Treg cells in the Gut-Associated Lymphoid Tissue (GALT) [41,46,47]. Microbial dysbiosis can lead to increased Th17 and IL-17A+ T cells, while reducing Treg cell production, leading to immune activation and inflammation [41,43,46] (Figure 4). Additionally, there is evidence that immune cells may migrate from the intestine to the eye, with T cells originating from the gut being detected in the eyes of EAU mice [48].

Studies in EAU mice have shown that broad-spectrum antibiotics can alter the microbiota, reducing *Firmicutes* and *Bacteroidetes* while increasing *Gammaproteobacteria*. This change in microbiota composition reduces uveitis severity by increasing Treg cells in lymphoid tissues and the eye [49], reducing bacterial diversity and IL-17A+ T cells in the gut [43]. Although greater microbial diversity is typically associated with a healthy microbiome, in the context of uveitis, this reduction in diversity likely reflects a selective decrease in pro-inflammatory bacterial species.

### 6.3. Reduction in Anti-Inflammatory Microbial Metabolites

**Figure 5:**
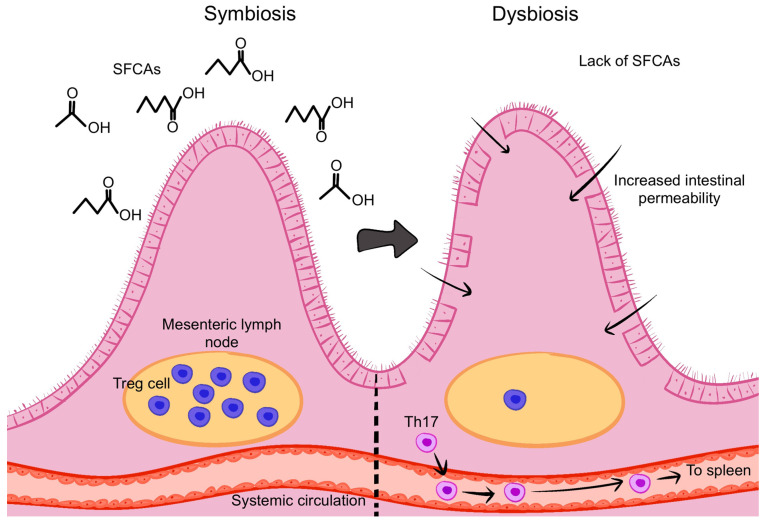
Short-chain fatty acids (SCFAs) produced by gut microbiota help maintain intestinal barrier function, promote Treg cells, and reduce Th17 cell migration. Image created with Procreate, Savage Interactive Pty Ltd., Version History: 5.3.5.

Gut microbes produce thousands of metabolites, with short-chain fatty acids (SCFAs) like butyrate, propionate, and acetate being the most common. These are byproducts of fiber fermentation by gut microbiota [41]. SCFAs play a crucial role in maintaining intestinal barrier integrity, promoting Treg cell populations in colonic lymph nodes, and limiting the movement of Th17 cells to the spleen [41,48] (Figure 5).

In C57BL/6J EAU mice, oral propionate treatment reduced the severity of uveitis by enhancing Treg cells in the intestinal lamina propria and mesenteric lymph nodes [48]. Similarly, intraperitoneal injection of butyrate alleviates EAU in these mice by reducing CD45+-infiltrating cells, monocyte subsets, and pro-inflammatory cytokines in ocular tissue [50].

Additionally, a study found that people with acute anterior uveitis exhibited significantly higher levels of seven specific metabolites in fecal DNA, including 6-deoxy-D-glucose, linoleic acid, and N-acetyl-beta-D-mannosamine in the gut microbiota, compared to healthy controls. While these metabolites may have an inflammatory effect, their precise role in acute anterior uveitis is still not fully understood [51].

### 6.4. Loss of Intestinal Barrier Integrity/Increased Intestinal Permeability

The intestinal epithelium, mucus layers, and antimicrobial peptides form key protective barriers against pathogenic microbes [31]. Gut commensal microbes produce both anti-inflammatory metabolites, such as SCFAs, and pro-inflammatory proteins, which influence the integrity of the colonic wall [31,52]. Dysbiosis disrupts these barriers, increasing intestinal permeability and allowing harmful microbes and their byproducts, such as lipopolysaccharides and beta-glucans, to enter the bloodstream and reach the eye [31,46,53].

**Figure 6:**
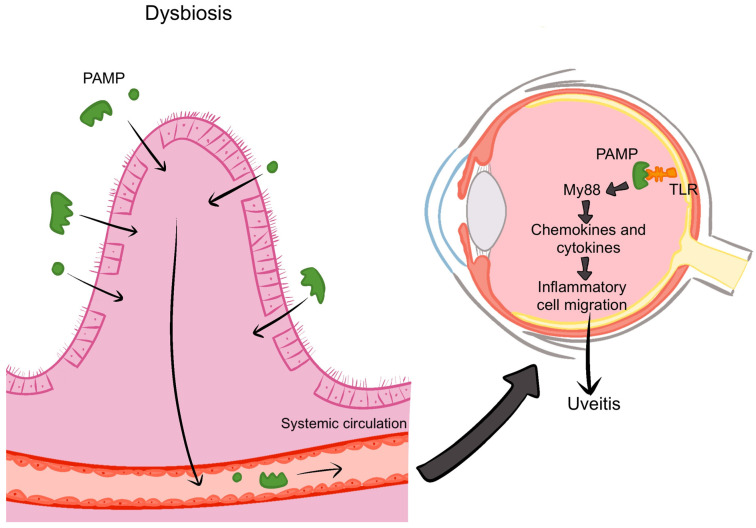
Dysbiosis results in increased intestinal permeability, allowing microbiota and their products to enter the blood circulation and reach the eye. Toll-like receptors (TLRs) on retinal pigment epithelium and uveal antigen-presenting cells recognize pathogen-associated molecular patterns (PAMPs), triggering a signaling cascade through Myeloid Differentiation Factor 88 (MYD88) and nuclear factor kappa-B (NF-κB). This cascade leads to the production of cytokines and chemokines that disrupt the blood–retinal and blood–aqueous barriers, promoting the migration of immune cells into the eye and resulting in uveitis [9]. Image created with Procreate, Savage Interactive Pty Ltd., Version History: 5.3.5.

The resulting breach in the gut’s defense mechanisms enables microbiota and their products to circulate through the bloodstream and access the eye. Toll-like receptors (TLRs) on retinal pigment epithelium and uveal antigen-presenting cells detect pathogen-associated molecular patterns (PAMPs), initiating a signaling cascade via Myeloid Differentiation Factor 88 (MYD88) and nuclear factor kappa-B (NF-κB). This cascade promotes the release of cytokines and chemokines, which compromise the blood–retinal and blood–aqueous barriers. This disruption facilitates immune cell migration into the eye, contributing to the development of uveitis [9] (Figure 6).

Research in EAU mice has demonstrated that increased intestinal permeability is associated with both an earlier onset and greater severity of uveitis. For example, mice immunized with a retinal protein peptide (interphotoreceptor retinoid-binding protein [IRBP]) in combination with *Mycobacterium tuberculosis* (MTB) exhibited higher intestinal permeability, contributing to uveitis onset and the degree of intestinal inflammation correlated with the severity of ocular inflammation [53].

In patients with ankylosing spondylitis, studies have shown a compromised gut–vascular barrier, altered intestinal tight junction expression, and increased serum lipopolysaccharides [38,54]. While ankylosing spondylitis is associated with uveitis, there are currently no studies documenting changes in gut architecture in ankylosing spondylitis patients with uveitis.

## 7. Gut Microbiome in Systemic Diseases Associated with Uveitis

Autoimmune uveitis is associated with many systemic diseases, including ankylosing spondylitis [55,56,57], inflammatory bowel disease [58,59,60], psoriasis [61], sarcoidosis [62,63,64,65], Vogt–Koyanagi–Harada disease [66,67], and Behçet’s disease [68,69,70,71,72,73,74]. There is increasing evidence for dysbiosis in these systemic illnesses. On a broader scale, if microbiome profiles for specific uveitis subtypes can be developed, population-wide screening could help identify individuals at higher risk of developing uveitis.

### 7.1. Ankylosing Spondylitis

Significant differences have been observed in the gut microbiome between patients with ankylosing spondylitis and healthy controls, yet no specific or consistent microbiome profile has been identified for ankylosing spondylitis [55]. Some studies, particularly in Chinese cohorts, have reported a reduction in *Bacteroides*, while an Italian study found decreased levels of *Veillonellaceae* and *Prevotellaceae* in patients with ankylosing spondylitis [55]. Morandi et al. [56] discovered that patients with HLA-B27-associated uveitis had elevated levels of *Eubacterium ramulus* and upregulated lipid biosynthesis pathways compared to controls. These findings further support a link between microbiome dysbiosis and uveitis.

More recently, a large-scale study has utilised Mendelian randomization to address the causality question, by examining the risk of developing ankylosing spondylitis with specific gut microbiota. *Bacteroides vulgatus* is associated with higher risk (odds ratio 1.55) and eleven bacterial traits also showed increased risk, with three showing reduced risk [57].

### 7.2. Inflammatory Bowel Disease

Ulcerative colitis and Crohn’s disease share similar gut microbiome characteristics [58]. In both conditions, there is a reduction in *Faecalibacterium prausnitizii* and *Firmicutes* (including *Clostridium*), while *Proteobacteria* and mucolytic *Ruminococcus* bacteria are more prevalent [58]. Additionally, other bacteria linked to the onset of inflammatory bowel disease include species of *Campylobacter*, *Salmonella*, and *Listeria monocytogenes* [59]. Interestingly, unlike ankylosing spondylitis, a Mendelian randomization study did not show a mediating role for gut microbiota in inflammatory bowel disease development [60].

### 7.3. Psoriasis

Multiple studies using 16S rRNA sequencing of the gut microbiome in patients with psoriatic arthritis have found significant differences in the gut microbiome compared to controls, with the most consistent findings being an increase in the mucolytic *Ruminococcus* bacteria, *Lachnospiraceae*, and *Firmucates* phylum [61].

### 7.4. Sarcoidosis

There is limited evidence connecting the gut microbiome to sarcoidosis. However, a study examining the blood microbiome of seven sarcoidosis patients found a significant decrease in *Bradyrhizobium*, *Pseudomonas*, *Comamonas*, and *Novosphingobium*, while *Veillonella* and *Prevotella* were significantly increased compared to healthy controls [62]. There have also been alterations observed in the lung fungal and bacterial microbiome in sarcoidosis with increased *Aspergillus* and lower bacterial diversity in sarcoid [63,64]. Another study observed increased *Atopobium* and *Fusobacterium* species within the sarcoid lung microbiome [65].

### 7.5. Vogt–Koyangi–Harada Disease

Microbiome analysis of fecal samples from active, untreated patients with Vogt–Koyanagi–Harada (VKH) revealed distinct differences compared to healthy individuals. Li et al. [66] found that VKH patients had higher levels of *Stomatobaculum*, *Pseudomonas*, and *Lachnoanaerobaculum*, while *Gordonibacter* and *Slackia* were depleted. Similarly, Ye et al. [67] reported increased levels of Paraprevotella and reduced levels of *Clostridium*, *Bifidobacterium*, and *Methanoculleus* in VKH. Additionally, Ye et al. [67] identified two microbial marker profiles that could potentially distinguish VKH patients from healthy controls [67].

### 7.6. Behçet’s Disease

Ye et al. [68] found that the microbiome composition of patients with active, untreated Behçet’s disease with ocular involvement differs significantly from that of healthy individuals. In Behçet’s patients, there was an increase in *Bilophilia*, *Parabacteroides*, and *Paraprevotella*, while levels of *Clostridium*, *Methanoculleus*, and *Methanomethylophilus* were reduced. A further study comparing untreated Behçet’s uveitis to Fuchs’ uveitis syndrome and healthy controls observed differences in both gut microbial composition and also in metabolome, with increased *Fusicatenibacter* in Behçet’s disease [69]. Interestingly, differences were also observed in the oral microbiome between Behçet’s patients and healthy controls. Other studies on the gut microbiomes of Behçet’s patients, including those without ocular involvement, reported similar findings, showing that Behçet’s has a distinct microbiome compared to healthy individuals [70,71,72]. Mendelian randomization population studies have observed increased risk of developing Behçet’s disease with *Streptococcaceae* and *Intestinibacter* abundance, whereas *Parasutterella*, *Lachnospiracease*, *Turicbacter*, and *Erysipelatoclostridium* were protective [73,74].

## 8. Gut Microbiome in Uveitis

The role of the microbiome in uveitis onset and disease course has been investigated via animal models, particularly in mice [43,49,53,75]. These studies have provided critical insights into how microbial communities influence intraocular inflammation and immune responses.

Germ-free EAU mice, which are raised in sterile environments and lack a microbiome, exhibit significant alterations in immune system development, including reduced numbers of IFN-γ and IL-17-expressing T cells and increased Treg cells in the eye-draining lymph nodes [75]. These germ-free EAU mice show markedly attenuated uveitis severity and reduced levels of T cells and macrophages in the retina compared to conventionally raised mice, suggesting that the presence of microbiota is essential for full activation of the immune response [75].

Broad spectrum antibiotics have been used to investigate the relationship between microbiota and uveitis. When administered one week prior to the induction of EAU, antibiotics significantly delay the onset and reduce the severity of spontaneous EAU [43], likely by disrupting microbial populations that promote pro-inflammatory immune responses. Additionally, oral administration of metronidazole and vancomycin, but not peritoneal administration, reduced intraocular inflammation and increased Treg induction in EAU mice [49]. Interestingly, the timing and duration of antibiotic treatment are critical. Prolonged antibiotic exposure (e.g., 10 weeks) prior to EAU induction leads to the loss of the protective effect observed with shorter treatment durations [76]. This suggests that chronic antibiotic use may induce dysbiosis or deplete protective microbial populations, negating any beneficial effect on immune regulation.

Other studies have observed alteration in the gut microbiome of EAU mice, including increased levels of *Clostridia* and *Lactobacillus* before the peak of uveitis [53]. In a more specific uveitis model, studies of HLA-B27 transgenic rats suggest that HLA-B27 expression affects the microbiome composition, increasing *Prevotella* spp. and decreasing *Rikenellaceae*, which may further influence disease progression [77].

Human studies investigating the microbiome’s role in autoimmune uveitis have provided valuable insights, though significant challenges remain. Unlike animal models, studying human populations is complicated by the inability to observe microbiome changes prior to the onset of disease, limiting our understanding of causality.

Patients with undifferentiated and immune-mediated uveitis show decreased diversity in both bacterial and fungal microbiota, with an increase in pro-inflammatory and opportunistic species [22,78]. One study involving an Indian cohort of 13 patients with non-infectious uveitis and 13 healthy controls found that uveitis patients had a reduced abundance and diversity of anti-inflammatory, butyrate-producing gut bacteria, including *Faecalibacterium*, *Lachnospira*, and *Ruminococcus*. In contrast, the uveitis group had higher levels of pro-inflammatory and pathogenic bacteria, such as *Prevotella copri* and *Streptococcus* [22]. Another study from Hyderabad revealed a reduced presence of gut fungal species with anti-inflammatory and anti-pathogenic properties in uveitis patients compared to healthy controls. At the same time, there was an increase in opportunistic fungal species, including *Candida albicans* and *Aspergillus gracilis* [78].

The microbiome in specific uveitis entities provides valuable insights into potential shared or distinct microbial patterns and their role in disease pathogenesis across different clinical subtypes. Li et al. [79] identified an increased abundance of eleven microbial species in patients with ankylosing spondylitis-associated uveitis, many of which are involved in SCFA production and the pro-inflammatory arachidonic acid metabolism pathway [79]. Similarly, Yasar et al. [72] observed an elevation in *Lachnospiraceae NK4A136* in the gut microbiota of patients with uveitis due to Behçet’s disease.

## 9. Oral Microbiome and Uveitis

Emerging evidence suggests that the dysregulation of the oral microbiome, or oral dysbiosis, may influence systemic inflammatory diseases, including uveitis. Akcalı et al. [80] observed significantly higher levels of *A. actinomycetemcomitans*, *F. nucleatum*, *S. oralis*, *A. naeslundii*, and *V. dispar* in gingival samples of patients with idiopathic uveitis compared to healthy controls. A small Korean study also found that patients with uveitic glaucoma had lower levels of *Lactobacillus* in their buccal microbiome compared to those with open-angle glaucoma [81].

Behçet’s disease has been linked to the presence of periodontitis, and some authors have postulated that the declining incidence of Behçet’s disease worldwide may be linked to global improvements in oral health [82]. In patients with Behçet’s disease, several studies have reported alteration of the oral microbiome, with elevated levels of *Streptococcus sanguinis* in the oral mucosa, along with higher levels of *Veillonella*, *Gardnerella*, *Lactobacillus*, *Atopobium*, *Peptoniphilus*, *Corynebacterium*, and *Staphylococcus* [83,84].

## 10. Ocular Microbiome in Uveitis

The understanding of the ocular microbiome is still in its early stages. One proposed mechanism by which the ocular microbiome may contribute to uveitis involves an imbalance in the intraocular microbiota, leading to the overgrowth of pathogenic microbes. These microbes are detected by resident ocular antigen-presenting cells (APCs), such as ocular dendritic cells. Through afferent lymphatic vessels, APCs migrate to the nearest draining lymph nodes, where they are recognized by CD4+ T cells. Activated retina-specific T cells then migrate into the eyes, where they secrete pro-inflammatory cytokines and chemokines. This can disrupt the blood–retina barrier and recruit additional inflammatory cells and mediators to the eyes, resulting in intraocular inflammation [9].

Although still under investigation, the intraocular microbiome has been implicated in the pathogenesis of uveitis in a few clinical studies. For instance, a study of 32 patients with Seasonal Hyperacute Panuveitis found a higher prevalence of *anelloviruses* in vitreous samples compared to controls [85]. Additionally, Li et al. [9] observed elevated levels of *Propionibacterium acnes* in the aqueous humor samples of patients with previous intraocular inflammation. Earlier studies also detected *Propionibacterium acnes* in granulomas of patients with ocular sarcoidosis, further suggesting its role in intraocular inflammation [86,87].

## 11. Barriers to Proving Causality: Key Challenges

Establishing a direct causal link between microbiome changes and autoimmune diseases presents several challenges. The human gut microbiome is both highly diverse and dynamic, with each individual hosting a unique microbiome influenced by factors such as genetics, age, environment, and systemic co-morbidities [20,88,89,90]. This variability complicates the identification of universal microbial patterns associated with disease [89,90]. Additionally, functional redundancy within the microbiome suggests that microbial composition alone may not fully explain the disease mechanism [20,90]. The lack of standardized methodologies, ranging from sample collection to sequencing techniques and bioinformatics analysis, adds another layer to the complexity of microbiome research [91].

In the context of uveitis, which is a heterogeneous condition with multiple etiologies, the microbiome’s role may vary across different subtypes with distinct pathophysiologies. This variability further complicates the identification of a single causal mechanism [47,92,93]. Additionally, individual differences in immune response, genetic susceptibility, and microbiome composition suggest that identical microbial changes may not lead to uveitis in all individuals [47,92].

New techniques such as NGS and metagenomics can significantly enhance our understanding of the microbiome’s role in uveitis. By leveraging these technologies, future studies can uncover the complex microbial dynamics and their mechanism of uveitis pathogenesis. This will not only improve our understanding of the microbiome’s influence but also guide the development of more targeted and personalized therapeutic strategies.

## 12. Advances in Microbiome Analysis Techniques

### 12.1. Next-Generation Sequencing and Metagenomics

NGS refers to a group of modern sequencing technologies that allow for rapid, high-throughput analysis of DNA and RNA. NGS can sequence millions of DNA fragments simultaneously, making it far more efficient and capable of analyzing complex genomes and transcriptomes in much greater detail compared to first generation sequencing technologies [94,95]. This capacity enables clinicians to detect mutations in patients’ genomes which may predispose to disease, such as cancer and uveitis, to help identify underlying genetic or immune-related factors contributing to disease susceptibility or progression [96,97,98].

Metagenomics is the study of genetic material recovered directly from environmental samples, enabling the analysis of the entire genetic makeup of a community of organisms (such as microbes) without the need to isolate or culture them individually, thereby providing a better insight into genetic diversity, environment, and local function [99]. This approach is particularly useful for studying complex microbial communities that cannot easily be cultured in laboratory settings, such as those found in the human gut. The advancement in metagenomics is in part attributed to the success of implementation of NGS, such as 16S rRNA gene sequencing and whole genome shotgun sequencing, which allow scientists to better understand the interaction between bacterial communities within the microbiome [100,101]. It can therefore enable us to create links between the taxonomic profiling and functional roles of the microbiome community [100,101,102].

While NGS and metagenomics have been utilized in various aspects of medical practice, their application in the field of uveitis has been limited. Thus far, the few proof-of-concept studies conducted were analyzing the causative pathogens in patients with infectious uveitis [98,103]. Vitreous samples were obtained from active inflammatory eyes, where conventional analysis was performed in addition to NGS [103] or metagenomics [98,104]. In many instances, the causative pathogen identified from sequencing correlates with conventional cultures and PCR techniques [98,103,104]. In addition to infectious uveitis, there are implications for NGS in the diagnosis of vitreoretinal lymphoma [105,106]. Vitreous biopsies obtained from patients with confirmed, or suspected, vitreoretinal lymphoma were sent for malignant cytology and NGS analysis. In these studies, NGS-based approach analysis illustrated high sensitivity compared to conventional diagnostic tests [105,106].

### 12.2. Non-Invasive Sampling Methods for Microbiome Studies

Non-invasive sampling methods for the microbiome are increasingly preferred due to their minimal discomfort while still providing reliable and valuable data. The ocular surface offers a unique opportunity to study the microbiome, as non-invasive techniques are easily accessible and can yield important insights into ocular surface diseases. Several methods have been employed to examine the microbiome in ophthalmology [107,108,109,110,111,112].

One such method is tear fluid collection using conjunctival swabs, filter papers, or Schirmer strips, which can help elucidate how microbial composition correlates with the severity of dry eye disease [107,108,109]. Another approach involved scraping the eyelid margin to assess the microbiome communities associated with conditions like blepharitis and meibomian gland dysfunction [110,111]. Additionally, impression cytology—a technique in which a small, sterile filter paper is gently pressed against the ocular surface to collect both cells and microbial material—can provide valuable insights into how the microbiome influences conjunctival epithelial cells, particularly in patients with allergic conjunctival disease [112].

### 12.3. Limitations on Microbiome Analysis

While NGS and metagenomics can greatly enhance our understanding of the microbiome, there are limitations with its current technology. One challenge is that these methods cannot distinguish whether sequences originate from live or dead pathogens [113]. Therefore, the presence of a pathogen does not imply that it is transcriptionally active.

In addition, with our current technology, there are limitations in identifying specific pathogens and determining whether they are integral to the natural flora or implicated in specific diseases [114]. Factors such as the quality of sample, DNA extraction methods, and the sequence platform utilized can all impact the detection of rare or low-density microbes, which may not be adequately captured by current methods [114,115].

Another limitation is the lack of understanding of the functional role of different microbiomes and their interaction in the environment [116]. Interpreting these results can be challenging due to the absence of standardized protocols for determining how the presence of a pathogen affects its environment [114,116]. Additionally, with multiple organisms present in a given microbiome, it can be difficult to differentiate how specific pathogens contribute to dysbiosis in the context of other microbial communities [117].

Newer methods are being studied [117,118] to help understand how the presence of specific microbe populations could play a functional role in their native environment.

## 13. Therapeutic Potential

Modification of the microbiome may provide new therapeutic targets for managing uveitis. This approach can be explored through various strategies, including the use of probiotics, prebiotics, antibiotics, and fecal microbial transplantation (FMT).

Probiotics and prebiotics, often combined with dietary modifications, aim to restore balance to the gut microbiome [27,28,29,119]. Probiotics work by correcting microbial dysbiosis, replenishing the gut with beneficial bacteria that help modulate the immune response. Prebiotics provide the gut with substrates like SCFAs that promote regulatory T cell activity and enhance intestinal barrier function. Antibiotic therapy, on the other hand, focuses on reducing systemic inflammation by eliminating pathogenic, pro-inflammatory bacteria [43,49,75,120]. FMT offers a more direct approach by introducing a diverse and stable microbial community directly into the gut, which may help modulate immune function and potentially reduce disease severity [121].

### 13.1. Probiotics

Probiotics are live organisms that can alter the gut microbiota when administered in sufficient quantities. Probiotics could offer a promising therapeutic avenue for the treatment of uveitis. By restoring microbial balance, modulating immune responses, and improving gut barrier function, probiotics could complement traditional therapies for uveitis and potentially reduce the need for long-term immunosuppressive treatments [10,16].

Probiotics, such as *Bifidobacterium* and *Lactobacillus* species, promote gut health in a variety of ways. Probiotics improve the gut integrity, produce anti-inflammatory SCFAs, and induce regulatory T cells [27,29]. Murine studies have shown that probiotics like *Lactobacillus* spp. and *Bifidobacterium bifidum* decrease inflammatory activity. In EAU, probiotic supplementation with a mixture of *Lactobacillus casei*, *Lactobacillus acidophilus*, *Lactobacillus reuteri*, *Bifidobacterium bifidum*, and *Streptococcus thermophilus* reduced disease severity [121]. Additionally, the live probiotic *Escherichia coli* Nissle 1917 (EcN) improved intestinal mucosal integrity and protected against uveitis development in mice [122,123].

While direct clinical trials focusing on probiotics for uveitis are limited, studies in other autoimmune conditions have shown promise. In inflammatory bowel disease, probiotics have been shown to reduce disease flares and promote remission [124].

Despite the promising potential of probiotics in treating uveitis, several challenges remain. One major hurdle is the variability in probiotic strains and their effects. Not all probiotics are equally effective, and the specific strains most beneficial for uveitis remain to be identified. Additionally, the optimal dosage, duration of treatment, and method of administration for probiotics in uveitis are still unclear. Another limitation is the lack of large-scale, randomized controlled trials focused specifically on uveitis. Therefore, more targeted research is needed to determine the precise role of probiotics in uveitis treatment, to determine the more effective probiotic strains, and the best delivery method.

### 13.2. Prebiotics

Diet can alter the microbiome. Prebiotics are dietary components that support the growth and activity of beneficial SCFA-producing microbes and suppress the growth of harmful pathogens [10,16]. Prebiotics are found in fiber-rich plant foods like unrefined cereals, fruits, vegetables, and legumes [28,29,119]. Supplementing with SCFAs like propionate [48,125] and butyrate [126] has been shown to reduce inflammation and disease severity in animal models of EAU, suggesting their potential as treatment options. SCFAs act by promoting the differentiation of Tregs, which are key to maintaining immune tolerance and preventing autoimmune reactions. Increased SCFA production also reduces the activation of pro-inflammatory cytokines, potentially alleviating the inflammation. In patients with Behçet’s disease, reduced butyrate production is linked to increased inflammation, and dietary interventions, including butyrate supplementation, may improve disease outcomes [70,126,127].

As with probiotics, there is currently a lack of clinical trials directly evaluating their impact on uveitis. Future studies are required to determine the ideal dosage, form, and duration of prebiotic supplementation for uveitis.

### 13.3. Other Dietary Interventions

#### 13.3.1. Caloric Restriction Diet

Caloric restriction may help alleviate autoimmune uveitis. Li et al. [128] used single-cell RNA sequencing to analyze cervical-draining lymph nodes in mice on caloric restriction diets, with or without experimental autoimmune uveitis (EAU). The results showed that caloric restriction increased regulatory T cells (Tregs), altered immune cell metabolism, reduced EAU symptoms, and downregulated inflammatory and glycolysis genes [128]. Flow cytometry confirmed that caloric restriction inhibited Th1 and Th17 proliferation while promoting Treg proliferation. Additionally, caloric restriction balanced CD4+ T cells by inhibiting the PI3K/AKT/c-Myc pathway and reducing GM-CSF in Th17 cells. These findings suggest that caloric restriction could be a potential therapeutic strategy for autoimmune diseases [128].

#### 13.3.2. Ketogenic Diet

A ketogenic diet is defined by its high-fat, low-carbohydrate content. Duan et al. [129] demonstrate that a ketogenic diet (KD) affects the composition and function of immune cells. KD promotes the differentiation of regulatory T cells (Tregs) and increases their abundance in healthy mice. When challenged with experimental autoimmune uveitis, KD reduces inflammation, decreases the pathogenic activity of CD4+ T cells, and restores the balance between Th17 and Treg cells [129]. Additionally, KD lowers the proportion of Th17 cells and raises the number of Treg cells in the retina [129]. Analysis of immune cells from both the retina- and cervical-draining lymph nodes (CDLN) shows that KD helps reduce the heightened inflammatory responses, particularly in CD4+ T cells in the retina [129].

### 13.4. Antibiotic Therapy

Antibiotics can impact the microbiome by depleting harmful bacteria that may contribute to systemic inflammation, thereby re-establishing a more favorable microbiome and positively impacting uveitis management.

The use of antibiotics has been shown to reduce ocular inflammation in EAU mice. Studies have used various antibiotics, including ciprofloxacin [75], and broad-spectrum antibiotics that include ampicillin, metronidazole, neomycin, and vancomycin (AMNV) [43,49,120], both individually [49,120] and in combination [43]. Notably, when antibiotics were used in combination, they more effectively reduced disease severity, suggesting that multiple bacterial species contribute to uveitis rather than a single microorganism being responsible [120]. Timing remains critical, however, with studies observing optimal benefit when utilized a week prior to induction of experimental uveitis, and limited-to-no benefit when used simultaneously with experimental uveitis induction, or with long-term therapy [43,76,114].

The overuse or misuse of antibiotics can, however, lead to antibiotic resistance [10], which poses significant challenges for long-term management. In contrast to broad-spectrum antibiotics, the use of IgA may selectively modulate the gut microbiota by binding to harmful bacteria like *E. coli* without affecting beneficial bacteria like *Lactobacillus casei* [130]. In experimental models of colitis, oral administration of IgA monoclonal antibodies successfully prevented disease development, offering a potential therapeutic option for inflammatory bowel disease [130,131].

The use of antibiotics in uveitis management requires careful consideration. While antibiotics can be beneficial for controlling infections and potentially promoting the growth of beneficial bacteria, their use must be weighed against the risks, including antibiotic resistance and the disruption of the microbiome. Such disruptions can contribute to dysbiosis, potentially exacerbating inflammation and worsening the condition. Further research is essential to better understand the role of antibiotics in modifying the microbiome and to develop strategies to minimize the adverse effects of antibiotic-induced dysbiosis.

### 13.5. Fecal Microbiota Transplant

FMT involves transferring processed fecal matter from a healthy donor into a patient’s gastrointestinal tract to restore a beneficial microbial balance. Initially gaining recognition for its effectiveness in treating recurrent *Clostridium difficile* colitis [132], FMT has since shown promise in improving disease control for patients with inflammatory bowel disease by helping to re-establish a more balanced and diverse gut microbiome [121].

When the microbiomes of patients with Behçet’s disease or VKH were transplanted into EAU models, it significantly increased uveitis severity and inflammatory markers [16,67,68]. A CD25 knockout mouse model lacking IL-2 signaling develops severe Sjögren’s syndrome with lacrimal gland atrophy and dry eyes, a phenotype worsened in a germ-free environment. Fecal microbiota transplantation from wild-type mice improved lacrimal gland pathology and reduced disease activity in these mice [133].

To date, there is a lack of human trials of FMT in uveitis. Additionally, the success of FMT can have significant interindividual variability [134], which can influence the effectiveness of the transplant. EAU models are encouraging, and further research is needed to assess the safety and efficacy of FMT in uveitis.

### 13.6. Immunomodulatory Drugs

Immunosuppressive drugs, such as disease-modifying anti-rheumatic drugs (DMARDs) have been shown to be effective in controlling inflammation in uveitis. There is emerging evidence to suggest these drugs can alter the gut microbiome [135,136].

Sulfasalazine, for example, has antibiotic properties, reduces vascular permeability, and helps improve joint disease in ankylosing spondylitis [137]. Similarly, immunosuppressive treatment with cyclosporine A in patients with VKH disease reduced dysbiosis and resolved intraocular inflammation [67].

In an EAU murine model, methotrexate at low doses decreased cellular immune responses and induced changes in the gut microbiome. Mycophenolate mofetil increased Tregs, reduced suppression of effector T cells, and caused distinct shifts in the intestinal microbiota. These findings suggest that the immunomodulatory effects of these drugs are linked to specific alterations in gut microbial composition [138].

Biological drugs like adalimumab and infliximab have demonstrated microbial modulation in diseases like Crohn’s disease [139,140] and ankylosing spondylitis [141], with responders showing a restoration of microbiome balance, marked by a decrease in harmful bacteria (e.g., *Proteobacteria*) and an increase in beneficial species (e.g., *Lachnospiraceae*).

## 14. Future Directions and Challenges

While this narrative review provides a comprehensive overview of the current understanding of the microbiome’s role in uveitis, there are some limitations. Although this study aimed for a broad and inclusive search, the selection of studies was subjective, potentially introducing bias, and the lack of primary data analysis means no quantitative conclusions can be drawn. Many studies are observational, which makes it difficult to establish causality, and the considerable variability in study designs can complicate efforts to generalize the findings. Moreover, the reliance on animal models and the limited number of human studies can make it challenging to directly apply these findings to clinical practice. The microbiome’s diversity and individual variability further complicate the identification of consistent patterns linked to uveitis. While the review discusses potential mechanisms, our understanding remains incomplete, and emerging technologies such as NGS are underrepresented in older studies. Overall, this narrative review offers valuable insights into the potential role of the microbiome in uveitis. Further research, especially more controlled, human-based, and mechanistic studies, will be essential to validate these findings and their clinical implications.

The future of microbiome research in uveitis holds significant potential, with several emerging trends and advancements likely to shape the field. The majority of work to date has shown associations between those with active disease and particular microbiome profiles. Future work will lean more into demonstrating causality, through the induction of disease in animal models via fecal transplantation, and to examining the impact of treatment on disease outcomes in humans [142]. We need to gain greater understanding of the microbiome’s role throughout the disease course via longitudinal studies, to assess differences in microbiome for disease induction compared to disease maintenance or recurrence in an already primed individual. There is also potential for microbiome profile to be incorporated into screening for future disease risk. This needs to be coupled with an improved understanding of microbiome changes throughout the life course, particularly at extremes of age. Breastfeeding, for example, is associated with a lower risk of developing juvenile idiopathic arthritis, possibly due to an altered microbiome [143], but little is known about the impact of infant microbiome on later inflammatory disease onset. Future microbiome research will also need to address how factors such as socioeconomic status, geography, and ethnicity influence microbiome composition and health outcomes.

The majority of microbiome research to date has been focused on the gut microbiome. There are other microbiomes of note, in particular the perioral, skin, and ocular microbiomes. These non-gut microbiomes may play critical roles in local and systemic immune responses, potentially influencing disease development and progression. Increased aqueous flare has been observed in individuals with periodontitis [144], and the perioral microbiome has been observed to be altered in idiopathic uveitis and in Behçet’s disease compared to healthy controls [80,83]. Whilst previously presumed to be sterile, new evidence has shown presence of bacteria or virus in the aqueous humor of those with sarcoidosis or previous ocular inflammation [85,86,87]. The virome, comprising the collection of viruses within the human body, represents an exciting frontier in microbiome research and its link to immune-mediated diseases like uveitis. While bacteria have been the primary focus in microbiome studies, the role of viruses is increasingly recognized for its potential influence on immune function and inflammation. A few studies have touched on the role of the virome in ankylosing spondylitis and rheumatoid arthritis [145,146], but the role of the virome in uveitis has yet to be explored.

Microbiome research has been expanding beyond genomics to include other “omics” approaches such as metatransciptomics, metabalomics, and proteomics [147]. By integrating these approaches, researchers can gain a deeper understanding into not only microbiome composition, but also its action: microbial gene expression, metabolite production, and how these impact host cell immune function.

Finally, considerable thought needs to be given to ethical considerations surrounding microbiome manipulation as we explore therapeutic interventions for uveitis. Microbiome-based treatments, such as FMT, raise concerns about safety, patient consent, and long-term effects on the host’s immune system. The potential for unintended consequences, such as the transfer of pathogenic microbes or the disruption of beneficial microbial communities, must be carefully considered. These unintended consequences could potentially include other diseases related to the microbiome, such as obesity, neuropsychiatric disorders, autoimmune diseases, or even malignancies. These risks arise from the complexity of the gut microbiome, as altering it through transplantation can have unforeseen sequelae [148].

## 15. Conclusions

The microbiome appears to play a pivotal role in the development and progression of uveitis by influencing immune responses and inflammatory processes. Recent research highlights its potential impact on disease onset, severity, and the treatment response. The development of microbiome-based diagnostics and therapeutics holds significant promise for personalized medicine, offering opportunities to detect early microbial imbalances and tailor interventions accordingly. However, despite these advancements, the complexity of the microbiome–uveitis axis calls for further research, particularly through large-scale longitudinal studies, to fully understand these interactions. Unlocking the potential of microbiome-targeted therapies could revolutionize the management of uveitis and offer novel approaches to controlling ocular inflammation and preventing vision loss.

## Figures and Tables

**Table 1:** The SUN Working Group Anatomic Classification of Uveitis.

Type	Primary Site of Inflammation	Includes
Anterior	Anterior chamber	Iritis, iridocyclitis, anterior cyclitis
Intermediate	Vitreous	Pars planitis, posterior cyclitis, hyalitis
Posterior	Retina or choroid	Focal/multifocal/diffuse choroiditis, chorioretinitis, retinochroiditis, retinitis, neuroretinitis
Panuveitis	Anterior chamber, vitreous, retina, or choroid	

Modified from Jabs et al. [5].

**Table 2:** The principle bacterial genera in a healthy oral cavity [34].

	Gram Positive	Gram Negative
Cocci	*Abiotrophia* *Peptostreptococcus* *Streptococcus* *Stomatococcus*	*Moraxella* *Neisseria* *Veillonella*
Rods	*Actinomyces* *Bifidobacterium* *Corynebacterium* *Eubacterium* *Lactobacillus* *Propionibacterium* *Pseudoramibacter* *Rothia*	*Campylobacter* *Capnocytophaga* *Desulfobacter* *Desulfovibrio* *Eikenella* *Fusobacterium* *Hemophilus* *Leptotrichia* *Prevotella* *Selemonas* *Simonsiella* *Treponema* *Wolinella*

## Supplementary Materials

The following supporting information can be downloaded at: https://www.mdpi.com/article/10.3390/microorganisms13020271/s1, Table S1: Commonly found microbial taxa in different body sites. References [36,149,150] are cited in the Supplementary Materials.
