# Supplementary material for: From Dysbiosis to Disease: The Microbiome’s Influence on Uveitis Pathogenesis
Source: Microorganisms. 2025 Jan 25;13(2):271. doi: 10.3390/microorganisms13020271 (PMC11857511; doi:10.3390/microorganisms13020271)
Supplement: Supplementary file 1 [file microorganisms-13-00271-s001.zip › microorganisms-3404362-supplementary.pdf]

Supplemental Table S1. Commonly found microbial taxa in different body sites

Body Site	Microbial Taxa
Oral cavity	*Streptococcus,*[1,2] *Prevotella,*[1,2] *Fusobacterium,*[1] *Moraxella,*[1] *Prophyromonas,*[1,2] *Candida,*[1] *Trichomonas,*[1] *Wolinella,*[1] *Entamoeba,*[1] *Propionibacterium*[2]
Eye	*Staphylococcus,*[3] *Streptococcus,*[3] *Propionibacterium,*[3] *Diphtheroid,*[3] *Micrococcus*[3]
Stomach	*Helicobacter,*[1,2] *Ralstonia,*[1] *Haemophilus,*[1] *Veillonella,*[1,2] *Streptococcus,*[1] *Prevotella,*[1] *Staphylocuccus,*[1] *Gemella,*[1] *Lactobacillus,*[2]
Intestine	*Lactobacillus,*[1,2] *Prevotella,*[1,2] *Escherichia,*[1,2] *Klebsiella,*[1,2] *Clostridium,*[1,2] *Bacteroides,*[1,2] *Streptococcus,*[1,2] *Veillonella,*[1] *Staphylococcus,*[1] *Prevotella,*[1] *Bacteroides,*[1] *Faecalibacterium,*[1,2] *Dorea,*[1,2] *Ruminococcus,*[1,2] *Blautia,*[1,2] *Collinsella,*[1,2] *Magasphaera,*[1,2] *Bifidobacterium,*[1,2] *Candida,*[1,2] *Enterobacter,*[2] *Streptococcus,*[2] *Fusobacterium,*[2] *Brachyspira,*[2] *Methylobacterium,*[2] *Akkermansia Saccaromyces,*[2] *Methanobrevibacter*[2]
Nasal cavity, oropharynx, Lungs	*Streptococcus,*[1,2] *Veillonella,*[1,2] *Rothia,*[1,2] *Prevotella,*[1,2] *Actinomyces,*[1] *Fusobacterium,*[1] *Prophyromonas,*[1] *Neisseria,*[1] *Staphylococcus,*[1,2] *Rothia,*[1] *Streptococcus,*[1] *Moraxella,*[1,2] *Leptotrichia,*[1,2] *Tropheryma,*[1,2] *Haemophilus,*[1] *Prevotella,*[1] *Corynebacterium,*[2] *Staphylococcus,*[2] *Dolosigranulum*[2]
Reproductive tract	*Lactobacillus,*[1,2] *Halomonas,*[1,2] *Atopobium,*[1,2] *Sneathia,*[1,2] *Cryptobacterium,*[1] *Eggerthella,*[1] *Aerococcus,*[1] *Peptoniphilus,*[1] *Fineglodia,*[1] *Gardnerella vaginalis,*[2]
Skin	*Corynebacterium,*[1,2] *Cutibacterium,*[1,2] *Micrococcus,*[1] *Haemophilus,*[1,2] *Streptococus,*[1,2] *Staphylococcus,*[1,2] *Pseudomonas,*[1,2] *Micrococcus,*[2] *Branhamella,*[2] *Streptophyta,*[2] *Micrococcineae,*[2] *Veillonella*[2]

1. Ahrodia T, Das S, Bakshi S, Das B. Structure, functions, and diversity of the healthy human microbiome. *Prog Mol Biol Transl Sci*. **2022**;191(1):53-82. doi: 10.1016/bs.pmbts.2022.07.003.

2. Das B. An introduction to human microbiome. *Prog Mol Biol Transl Sci*. **2022**;191(1):1-28. doi: 10.1016/bs.pmbts.2022.06.026.

3. Miller D, Iovieno A. The role of microbial flora on the ocular surface. *Curr Opin Allergy Clin Immunol*. **2009**;9(5):466-70. 787 doi: 10.1097/ACI.0b013e3283303e1b
